# Quantifying the shifts in physicochemical property space introduced by the metabolism of small organic molecules

**DOI:** 10.1186/1758-2946-5-S1-O12

**Published:** 2013-03-22

**Authors:** Johannes Kirchmair, Andrew Howlett, Julio Peironcely, Daniel S Murrell, Mark Williamson, Samuel E Adams, Thomas Hankemeier, Leo van Buren, Guus Duchateau, Werner Klaffke, Robert C Glen

**Affiliations:** 1 Unilever Centre for Molecular Sciences Informatics, Department of Chemistry, University of Cambridge, Lensfield Road, Cambridge, CB2 1EW, UK; 2 TNO Research Group Quality & Safety, P.O. Box 360, 3700 AJ Zeist, The Netherlands; 3 Leiden/Amsterdam Center for Drug Research, Leiden University, 2333 CC Leiden, The Netherlands; 4 Netherlands Metabolomics Centre, 2333 CC Leiden, The Netherlands; 5 Unilever R&D, 3133 AT Vlaardingen, The Netherlands

## 

**Figure 1:**
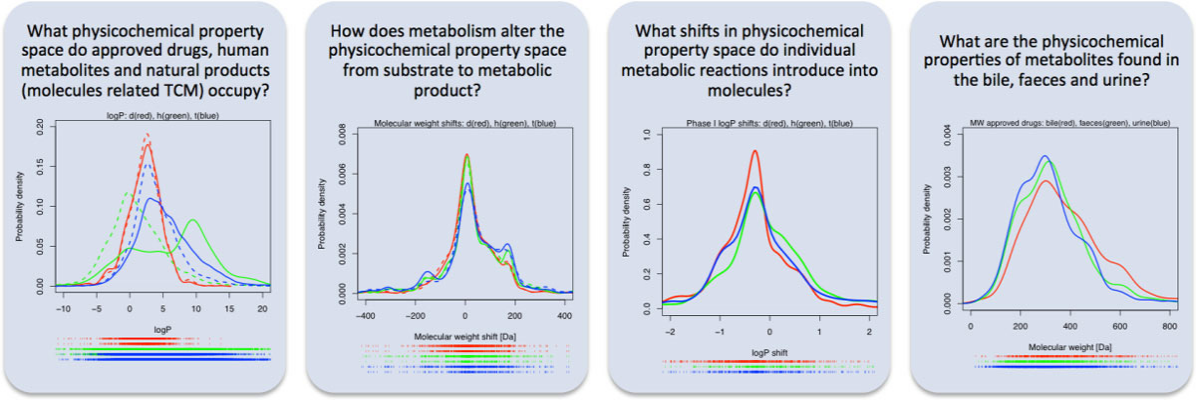
**Four important questions pertinent to the design and development of new molecules with favourable ADME properties addressed in this work**. d, approved drugs; h, human metabolites; t, molecules from traditional Chinese medicines; MW, molecular weight.

Understanding the metabolic fate of small organic molecules is of fundamental importance to the successful design and development of drugs, nutritional supplements, cosmetics and agrochemicals [1,2]. In the current study we investigated how the products of metabolism differ from their parent molecules by analysing a large dataset of experimentally determined metabolic transformations (Figure 1). This dataset was split into three specific chemical domains representing approved drug molecules, human metabolites and molecules from traditional Chinese medicines to allow individual analysis. We also quantified the impact of individual Phase I and Phase II metabolic reactions on calculated chemical descriptors using MetaPrint2D [3] and suggest new approaches to utilise metabolism for the design of drugs and cosmetics. The last section of this study investigates the properties of metabolites found in the bile, faeces and urine and analyses their commonalities and differences.
